# AZD1775 Increases Sensitivity to Olaparib and Gemcitabine in Cancer Cells with p53 Mutations

**DOI:** 10.3390/cancers10050149

**Published:** 2018-05-19

**Authors:** Xiangbing Meng, Jianling Bi, Yujun Li, Shujie Yang, Yuping Zhang, Mary Li, Haitao Liu, Yiyang Li, Megan E. Mcdonald, Kristina W. Thiel, Kuo-Kuang Wen, Xinhao Wang, Meng Wu, Kimberly K. Leslie

**Affiliations:** 1 Department of Obstetrics and Gynecology, Carver College of Medicine, The University of Iowa, Iowa City, IA 52242, USA; jianling-bi@uiowa.edu (J.B.); li_yujun@126.com (Yu.L.); shujie-yang@uiowa.edu (S.Y.); yuping-zhang@uiowa.edu (Yi.Z.); maryli1214@gmail.com (M.L.); haitaoliulht@163.com (H.L.); liyiyang1980@163.com (Y.L.); megan-e-mcdonald@uiowa.edu (M.E.M.); kristina-thiel@uiowa.edu (K.W.T.); xwang352@healthcare.uiowa.edu (X.W.); 2 Holden Comprehensive Cancer Center, The University of Iowa, Iowa City, IA 52242, USA; meng-wu@uiowa.edu; 3 High Throughput Screening Facility at University of Iowa (UIHTS), Iowa City, IA 52242, USA; kuo-kuang-wen@uiowa.edu; 4 Department of Biochemistry, Carver College of Medicine, Iowa City, IA 52242, USA; 5 Division of Medicinal and Natural Products Chemistry, Department of Pharmaceutical Sciences and Experimental Therapeutics, College of Pharmacy, The University of Iowa, Iowa City, IA 52242, USA

**Keywords:** p53, WEE1, PARP1, AZD1775, olaparib, gemcitabine

## Abstract

Tumor suppressor p53 is responsible for enforcing cell cycle checkpoints at G1/S and G2/M in response to DNA damage, thereby allowing both normal and tumor cells to repair DNA before entering S and M. However, tumor cells with absent or mutated p53 are able to activate alternative signaling pathways that maintain the G2/M checkpoint, which becomes uniquely critical for the survival of such tumor cells. We hypothesized that abrogation of the G2 checkpoint might preferentially sensitize p53-defective tumor cells to DNA-damaging agents and spare normal cells with intact p53 function. The tyrosine kinase WEE1 regulates cdc2 activity at the G2/M checkpoint and prevents entry into mitosis in response to DNA damage or stalled DNA replication. AZD1775 is a WEE1 inhibitor that overrides and opens the G2/M checkpoint by preventing WEE1-mediated phosphorylation of cdc2 at tyrosine 15. In this study, we assessed the effect of AZD1775 on endometrial and ovarian cancer cells in the presence of two DNA damaging agents, the PARP1 inhibitor, olaparib, and the chemotherapeutic agent, gemcitabine. We show that AZD1775 alone is effective as a therapeutic agent against some p53 mutated cell models. Moreover, the combination of AZD1775 with olaparib or gemcitabine is synergistic in cells with mutant p53 and constitutes a new approach that should be considered in the treatment of advanced and recurrent gynecologic cancer.

## 1. Introduction

Wild-type p53 enforces both G1/S and G2/M cell cycle checkpoints as a means to allow all cells to repair DNA before entering S and M. The G1/S checkpoint is enhanced by the induction of p21 transcription by p53, particularly in response to DNA damage, and the G2/M checkpoint is normally maintained by events downstream of p53, including CHK1-mediated phosphorylation of cdc25c and WEE1-mediated phosphorylation of cdc2. WEE1 is a member of the serine/threonine protein kinase family and is a key regulator of cell cycle progression. Thus, WEE1 is one of the most important G2 checkpoint regulating kinases and it prevents entry into mitosis in response to DNA damage. 

TP53 is the most frequently mutated gene tumorigenesis. p53 null cancer cells or those with single amino acid modifications are typically more aggressive and difficult to treat compared to tumor cells with wild-type p53 [1,2]. How do cells with defective or absent p53 repair DNA sufficiently and successfully progress through the cell cycle? DNA-damaged cells with defective p53 function rely heavily on the G2 checkpoint for survival, which is maintained by alternative signaling pathways in the absence of normal p53. Therefore, therapeutic abrogation of the G2 checkpoint might preferentially sensitize p53-defective tumor cells to DNA-damaging agents while sparing normal cells with intact p53 function [3].

AZD1775, previously known as MK1775, is a pyrazolo-pyrimidine derivative that is a selective ATP competitive small molecule inhibitor of the WEE1 kinase with effective checkpoint inhibitory activity [4,5]. Toxicity studies from a Phase I trial with AZD1775 suggested that it could be safely combined with a variety of chemotherapy agents to treat solid tumors [6]. Two such agents, the PARP1 inhibitor, olaparib, and the chemotherapeutic agent, gemcitabine, are of particular interest based upon previous studies and known activity in a variety of cancers [7,8].

Among the 17 members of the PARP family, PARP1 is one of the most abundant proteins involved in transcriptional control, maintenance of genomic integrity, DNA repair, and regulation of apoptotic and survival balance [9,10]. Tumor cells with defects in other DNA repair pathways often rely heavily on PARP activity to repair DNA. Hence, PARP1 inhibitors are of particular interest for the treatment of cancers with inherent defects in their DNA repair pathways, such as in BRCA1/2 [11]. Olaparib is one of the PARP1 inhibitors used in the treatment of gynecologic and other cancers [12,13]; however, standard chemotherapeutic agents remain a mainstay of therapy as well. One such chemotherapeutic agent is Gemcitabine (2′,2′-difluorodeoxycytidine), which is one of many non-platinum agents that exhibit activity in recurrent, platinum-resistant ovarian cancer [12]. Gemcitabine also shows activity in other tumor types including pancreatic cancer, where apoptosis occurs as a result of the downregulation of critical signaling pathways and interference with DNA repair [14]. Preclinical studies of WEE1 inhibitors in combination with cytotoxic agents suggest potential synergy, while clinical studies demonstrate encouraging antitumor patterns with manageable side effects [3,15]. 

In this study, the effect of AZD1775 as a single agent or in combination with olaparib or gemcitabine was investigated in gynecological cancer cells with the hypothesis that this strategy would be particularly effective against cells with mutated p53. As p53 mutations are a hallmark of the most aggressive endometrial and ovarian tumors, new strategies to treat these cancers are critical to improve patient care and therapeutic outcomes. 

## 2. Results

### 2.1. The WEE1 Inhibitor AZD1775 Increases Sensitivity to the PARP Inhibitor Olaparib

**Figure 1:**
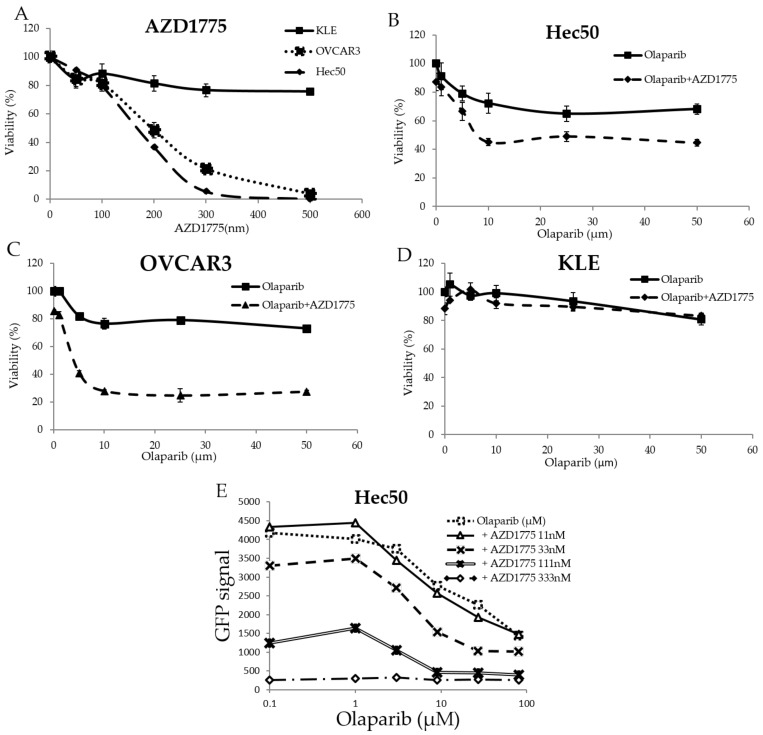
Effect of AZD1775 and olaparib on the viability of BRCA wild-type, p53 mutated gynecology cancer cells. (**A**) Sensitivity to AZD1775 in Hec50, KLE, and OVCAR3 cells was tested via WST-1 assay; sensitivity to olaparib alone or olaparib + AZD1775 was tested in cancer cell lines (**B**) Hec50, (**C**) OVCAR3, and (**D**) KLE; (**E**) the expression of the reporter, GFP, was detected in Hec50 cells treated with the combination of olaparib and AZD1775 at different concentrations.

In order to test the effect of WEE1 inhibition as a single intervention on gynecological cancer cells, the endometrial cancer cell lines, Hec50 and KLE, and the ovarian cancer cell line OVCAR3, which are wild-types for BRCA1/2, were treated for 72 h with different concentrations of the WEE1 inhibitor AZD1775. Hec50 cells represented the serous form of poorly-differentiated endometrial cancer and are null for p53 owing to a disrupting mutation in intron 6. KLE cells represented poorly-differentiated endometrial tumor cells that harbor the frequently-occurring R175H p53 mutation, which inhibits normal p53 DNA binding function resulting in the loss of tumor suppressor activity and a gain of oncogenic activity. OVCAR3 cells represented high grade serous ovarian cancers with the oncogenic mutation R248Q. As shown in Figure 1A, Hec50 cells and OVCAR3 cells were more sensitive to AZD1775 alone compared to KLE cells. 

Based upon the IC50 calculations from Figure 1A, AZD1775 concentrations at 50, 100, and 200 nm were selected to treat Hec50, KLE, and OVCAR3 cells in combination with different concentrations of olaparib. As shown in Figure 1B–D, AZD1775 significantly increased the sensitivity of Hec50 and OVCAR3 cells to olaparib, but not KLE cells, which remained relatively resistant to either AZD1775 alone or in combination with olaparib. 

To further study the effect of adding AZD1775 on olaparib sensitivity, Hec50 cells engineered to express the H2B-GFP reporter gene as a marker for proliferation were established and tested. As shown in Figure 1E, AZD1775 concentrations of 33, 111, and 333 nM showed a reduction in cell proliferation when compared to olaparib alone. Synergy associated with the most active combination was calculated using the Bliss (4.252) and HAS score (7.78) methods. 

### 2.2. Olaparib Combined with AZD1775 Increases Cell Death

**Figure 2:**
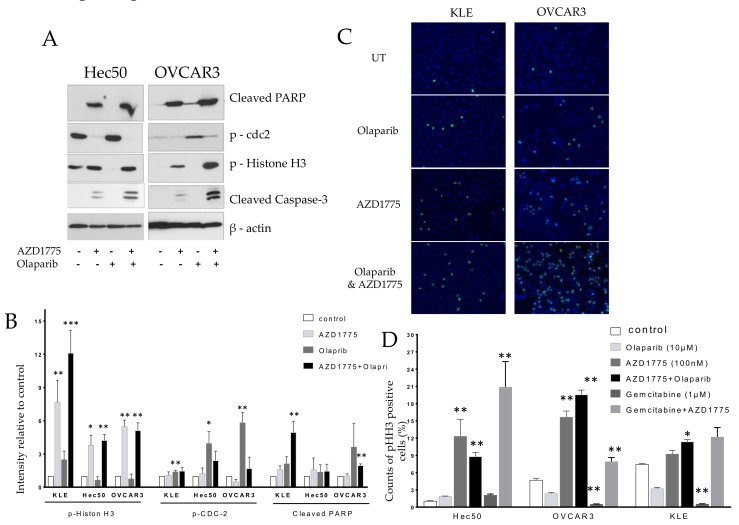
Effect of AZD1775 and olaparib treatment on the expression of apoptotic and mitotic biomarkers. (**A**) Antibodies against cleaved-PARP, cleaved-Caspase-3, phospho-histone H3 Ser10, and phosphorylated Cdc2 at Tyr15 were tested in Hec50 and OVCAR3 cells treated with AZD1775, olaparib, or the combination. “+” indicated cells treated with AZD1775 or Olaparib. “−“indicated cells untreated with AZD1775 or Olaparib. (**B**) Densitometric quantification of the intensity of indicated proteins relative to untreated control after normalization to loading control β-actin; data are representative of three independent experiments. (**C**) KLE and OVCAR3 cells in mitosis were indicated by the expression of mitosis biomarker phospho-Histone H3 at Ser10 (pHH3) after immunofluorescence staining (green color) and nuclei with DAPI. (**D**) Densitometric quantification of the percentage of phospho-histone H3 Ser10 positive cells from three repeated experiments; * *p* < 0.05, ** *p* < 0.01, *** *p* < 0.001; treatment vs. control.

Hec50 and OVCAR3 cells were treated with AZD1775, olaparib, or the combination of AZD1775 with olaparib for 24 h, followed by analysis of cell lysates via western blot. The combination of olaparib- and AZD1775-enhanced apoptosis was indicated by the upregulation of cleaved-PARP and cleaved-Caspase-3. The apoptotic effects of the combination were most notable in mitosis, as indicated by the upregulation of the mitosis biomarker, p-Histone H3 (pHH3); suggesting that treated cells were unable to successfully transit through M. In contrast, olaparib alone, which was active mostly in S phase, decreased the number of mitotic cells as shown by a reduction in pHH3 (Figure 2A). Confirmation of the induction of p-Histone H3 with combinatorial treatment is shown by immunostaining in Figure 2B.

### 2.3. AZD1775 Increases Sensitivity to Gemcitabine 

**Figure 3:**
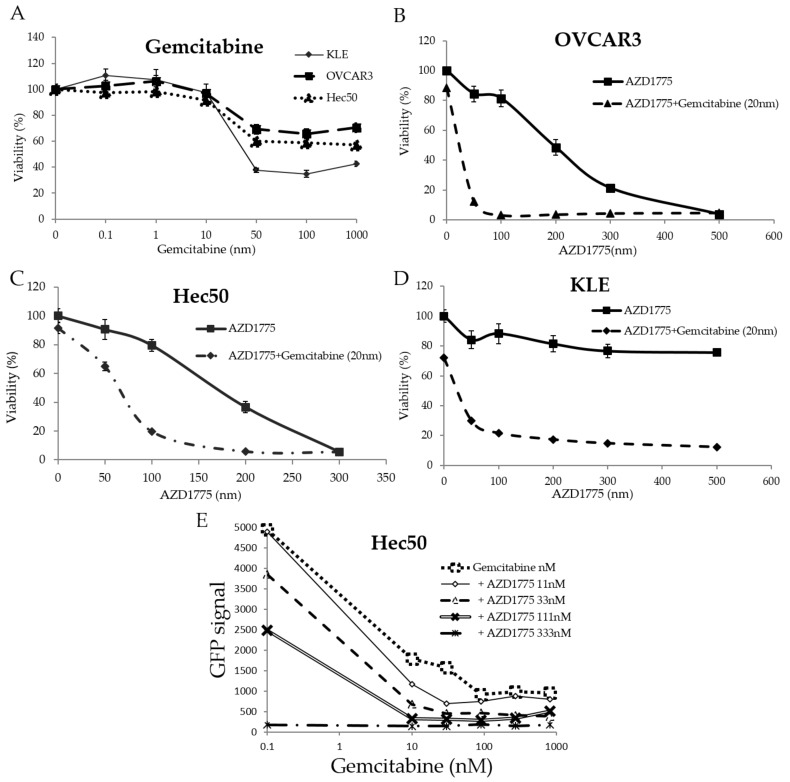
Effect of gemcitabine and AZD1775 on cell viability. (**A**) Sensitivity to gemcitabine as a single agent was tested by WST-1 in Hec50, KLE, and OVCAR3 cells. Sensitivity to AZD1775 alone or gemcitabine in combination with AZD1775 was tested in (**B**) OVCAR3; (**C**) Hec50; and (**D**) KLE cells. (**E**) The expression of the reporter GFP as a marker for proliferation was detected in Hec50 cells treated with combinations of gemcitabine and AZD1775 at increasing concentrations.

Experiments were next carried out to determine the effectiveness of combining the WEE1 inhibitor AZD1775 and gemcitabine, with hopes of addressing KLE cell resistance to the AZD1775 + olaparib combination. Each of the cell lines demonstrated modest sensitivity to gemcitibine alone at increasing concentrations from 0.1 nM to 1000 nM (Figure 3A). Using 20 nM gemcitabine in combination with varying doses of AZD1775 resulted in significant cell death in all cell lines including OVCAR3, Hec50 and KLE (3B, 3C and 3D). After testing the impact of fixed concentrations of AZD1775 (33, 111, and 333 nM) on the gemcitabine IC_50_, it became clear that WEE1 inhibition greatly increased cell sensitivity to low doses of gemcitabine (Figure 3E). The synergy resulting from combinatorial treatment was calculated to be 15.115 by the Bliss method and 15.155 by the HSA method. 

### 2.4. Gemcitabine and AZD1775 Combination Increases Apoptosis

**Figure 4:**
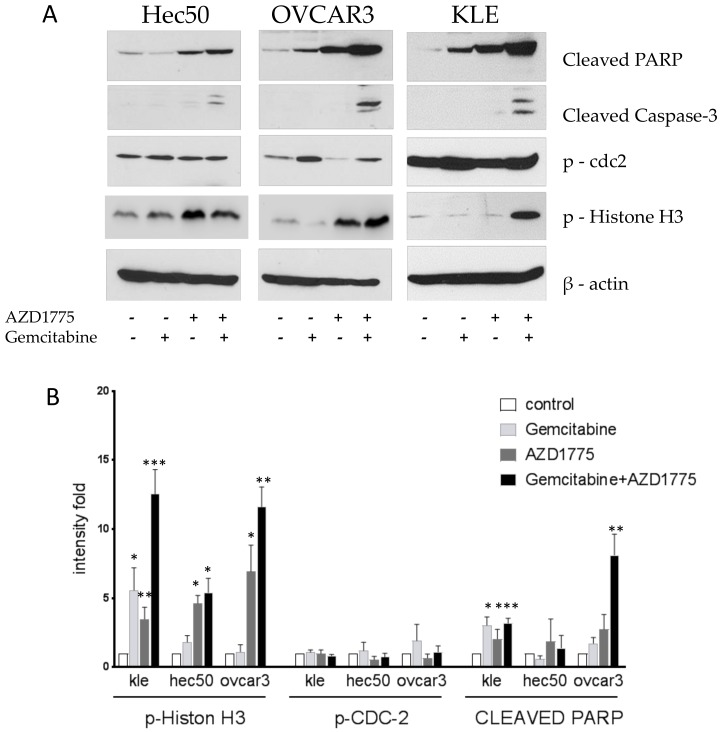
Effect of AZD1775 and gemcitabine treatment on the expression of apoptotic and mitotic biomarkers. (**A**) Antibodies against cleaved-PARP, cleaved-Caspase-3, phospho-histone H3 Ser10, and phosphorylated Cdc2 at Tyr15 were evaluated in Hec50, OVCAR3, and KLE cells treated with AZD1775, gemcitabine, or the combination. “+” indicated cells treated with AZD1775 or Olaparib. “−“indicated cells untreated with AZD1775 or Olaparib. (**B**) Densitometric quantification of the intensity of indicated proteins relative to untreated control after normalization to loading control β-actin; data are representative of three independent experiments.

Cells were treated with 1 μM gemcitabine for 8 h followed by the addition of 100 nM AZD1775 for an additional 16 h. Cells were then harvested, proteins were extracted, and Western blotting was performed. The combination of gemcitabine and AZD1775 induced the expression of two apoptosis biomarkers, cleaved-PARP and cleaved-Caspase-3 (Figure 4). Gemcitabine alone reduced the expression of p-Histone H3, reflective of its activity in S. Contrasting this, the combination treatment induced the expression of p-Histone H3, reflective of the enhanced cell killing in M when the G2/M checkpoint was abrogated by WEE1 inhibition (Figure 4).

## 3. Discussion

Due to the aggressive nature of mutant p53 cancer cells, better treatment options are needed to improve outcomes. These studies were performed in cells with mutated p53 to assess whether new combinations of agents that included the WEE1 inhibitor AZD1775 could be employed to enhance cell killing. Mutations in p53 are of various types and can be broadly categorized as either loss of function (p53 null, as seen in Hec50 cells) or gain of function, which are oncogenic mutations caused by single amino acid alternations typically in the DNA binding domain of p53 (as in KLE and OVCAR3 cells). Regardless of the type of p53 mutation, one can generalize that the regulation of cell cycle checkpoints is disrupted in the absence of normal p53 function. Hence, we hypothesized that treatments that capitalize on the changes in cell cycle resulting from mutations in p53 may show promise in such tumors.

The cell cycle transition from G2 into mitosis (M) is ultimately regulated by a phosphorylation event at tyrosine 15 of cdc2, which is reversibly modified by kinases WEE1 and MYT1, and the protein phosphatase cdc25. Transcription of cdc25 is suppressed by wild-type p53, resulting in constitutive phosphorylation of cdc2 and enforcement of the G2/M checkpoint. WEE1 inhibition reverses the phosphorylation of cdc2, thereby opening the G2/M checkpoint. Cells with mutated p53 depend mostly on the G2/M checkpoint to repair DNA because mutations in p53 result in the loss of the G1/S checkpoint due to the loss of p21 transcription activation by p53 in response to DNA damage. p53-mediated transcription activation of p21 plays a critical role as a CDK2 inhibitor at G1/S checkpoint in response to DNA damage [16]. Therefore, abrogation of the G2/M checkpoint, by inhibiting the critical kinase, WEE1, would enhance the therapeutic effectiveness of cytotoxic agents, such as olaparib and gemcitabine, and would be a successful strategy even in p53 mutated cells. 

The PARP1 inhibitor, olaparib, has been approved to treat breast and ovarian cancer patients who have germline mutations in BRCA1/2. However, even in BRCA wild-type cells, a PARP inhibitor may kill cancer cells, particularly when combined with other drugs. AZD1775 has been shown to enhance the effectiveness of olaparib in ovarian cancer cells with wild-type BRCA [14]. It has also been shown to potentiate the therapeutic response of cells to gemcitabine and cisplatin, a mechanism that involves overriding the G2/M checkpoint so that cancer cells with damaged DNA, as a result of chemotherapy, to prematurely enter M and die in mitotic catastrophe [17]. Hence, various preclinical and clinical studies have confirmed that AZD1775 has the potential to increase tumor sensitivity to chemotherapy [7,8]. The additional insight that such therapy may be particularly useful in treating tumors with mutated p53 derives from the studies we have presented above. Most notably, our data indicated that cells may selectively be more sensitive to different treatment combinations. For example: KLE endometrial cancer cells, which harbor the oncogenic p53 mutation R175H, were resistant to AZD1775 + olaparib but sensitive to AZD1775 + gemcitabine, whereas both combinations were effective in Hec50 and OVCAR3 cells. Further studies are required to assess the biomarkers of sensitivity to each regimen. Furthermore, AZD1775 may induce toxicity through double-stranded DNA breaks in addition to regulating the G2/M checkpoint as reported in colorectal cancer cells [18].

In summary, preclinical studies in cell models with various forms of mutations in p53 demonstrated that WEE1 inhibition in addition to either olaparib or gemcitabine was synergistic and resulted in significant cell death in mitosis. Tumors with mutant p53 are notoriously difficult to treat, so it is encouraging that this strategy shows promise. We propose that future clinical trials be designed to confirm our preclinical findings. 

## 4. Materials and Methods 

### 4.1. Cell Culture

Human endometrial cancer cell lines KLE and OVCAR3 were purchased from ATCC. Hec50 cells were a gift from Dr. Erlio Gurpide, New York University. KLE and OVCAR3 cells were maintained in RPMI1640 medium supplemented with 10% fetal bovine serum and 1% penicillin/streptomycin; Hec50 cells were maintained in DMEM supplemented with 10% fetal bovine serum and 1% penicillin/streptomycin (all from Gibco BRL, Carlsbad, CA, USA). Cells were incubated at 37 °C in a humidified atmosphere of 5% CO_2_. 

H2B-GFP expressing Hec50 cells were established after transfection with H2B-GFP expression plasmids, selected in Blasticidin, and sorted for GFP cells by flow cytometry. GFP expression was used as a reporter for cell proliferation.

Statistical analyses of synergy associated with combinatorial treatment were performed using the Bliss and HSA methods [19,20]. 

### 4.2. Drug Treatment and Cell Viability Assays

AZD1775, olaparib, and gemcitabine (purchased from Selleck Chemicals, Houston, TX, USA) were resuspended in Dimethyl sulfoxide (DMSO). At a concentration of 1 × 10^4^ cells/well, KLE and OVCAR3 cells were seeded in conventional 96-well plates with 100 μL of medium and allowed to adhere overnight. Cells were then exposed the following day to drug treatments for 72 h, and cell proliferation was analyzed using a WST-1 kit (Sigma, St. Louis, MO, USA). The WST-1 assay is a colorimetric test based on cleavage of the tetrazolium salt WST-1 into orange formazan by mitochondrial dehydrogenases in viable cells. The result was reported as a percentage of viable cells in the treatment arms compared to the control arm of each experiment. Each condition was performed in three replicated wells, and the data represent three independent experiments.

### 4.3. Western Blotting

Following treatment, cells were solubilized in cold *Nonidet P-40* (NP-40) cell lysis buffer (150 mM NaCl, 50 mM Tris/HCl, pH 7.4, 1% NP-40 with a protease and phosphatase inhibitor cocktail from ThermoFisher Scientific (Waltham, MA, USA) and then sonicated to release nuclear proteins. Lysates were boiled in a 2× Laemmli sample buffer for 5 min before they were subjected to SDS-PAGE, transferred onto nitrocellulose membranes, and immunoblotted with specific primary and horseradish peroxidase (HRP)-conjugated secondary antibodies. All antibodies were diluted 1:1000. The intensities of the reaction products were assessed with the Enhanced Chemiluminescence System (Pierce). Data were quantified by densitometry using NIH Image J, normalized to loading control, and the intensity calculated relative to untreated control, which was set at 1. 

### 4.4. Antibodies

Antibodies to p53, cleaved-PARP, cleaved-Caspase-3, phospho-cdc2 and phospho-Histone H3 were purchased from Cell Signaling Technology (CST); β-actin antibodies were obtained from Sigma. 

## 5. Conclusions

Treatment with the Wee1 inhibitor, AZD1775, alone or in combination with olaparib was effective as a therapeutic strategy against some p53 mutated cell models. The combination of AZD1775 with gemcitabine was synergistic in all tested cell models with mutant p53. Therefore, the combination of AZD1775 with gemcitabine is promising for the treatment of advanced and recurrent gynecologic cancer with p53 mutations and warrants future investigation in clinical trials. 

## Abbreviations

G1: the cell cycle G1 phase; S: the cell cycle DNA synthesis phase; G2: the cell cycle G2 phase; M: the cell cycle mitosis phase; BRCA1: breast cancer type 1 susceptibility protein.
